# Discovery of *N*-(Naphtho[1,2-*b*]Furan-5-Yl) Benzenesulfonamides as Novel Selective Inhibitors of Triple-Negative Breast Cancer (TNBC)

**DOI:** 10.3390/molecules23030678

**Published:** 2018-03-16

**Authors:** Ya Chen, Yong Tang, Beibei Mao, Wenchao Li, Hongwei Jin, Liangren Zhang, Zhenming Liu

**Affiliations:** 1 State Key Laboratory of Natural and Biomimetic Drugs, School of Pharmaceutical Sciences, Peking University, Beijing 100191, China; yachen@pkuddc.com (Y.C.); beibeimao@pkudddc.com (B.M.); wenchaoli@bjmu.edu.cn (W.L.); jinhw@bjmu.edu.cn (H.J.); 2 Beijing Shenogen Biomedical Co., Ltd, Beijing 102206, China; tang-yong@hotmail.com

**Keywords:** triple-negative breast cancer, three-dimensional similarity search, virtual screening, selective inhibitors

## Abstract

Any type of breast cancer not expressing genes of the estrogen receptor (ER), progesterone receptor (PR), or human epidermal growth factor receptor 2 (HER2) is referred to as triple-negative breast cancer (TNBC). Accordingly, TNBCs do not respond to hormonal therapies or medicines targeting the ER, PR, or HER2. Systemic chemotherapy is therefore the only treatment option available today and prognoses remain poor. We report the discovery and characterization of *N*-(naphtho[1,2-*b*]furan-5-yl)benzenesulfonamides as selective inhibitors of TNBCs. These inhibitors were identified by virtual screening and inhibited different TNBC cell lines with IC_50_ values of 2–3 μM. The compounds did not inhibit normal (i.e. MCF-7 and MCF-10A) cells in vitro, indicating their selectivity against TNBC cells. Considering the selectivity of these inhibitors for TNBC, these compounds and analogs can serve as a promising starting point for further research on effective TNBC inhibitors.

## 1. Introduction

Breast cancer is the most common malignancy and second leading cause of cancer death among women in the United States [1]. As in most countries, breast cancer is the most common cancer in Chinese women today. Cases in China account for 12.2% of all newly diagnosed breast cancers and for 9.6% of all deaths from breast cancer worldwide [2]. Based on DNA microarray expression profiling, breast cancers can be classified into six different subtypes [3,4,5,6,7,8]: luminal A, luminal B, human epidermal growth factor receptor-2 (HER2)-overexpressing, normal breast tissue-like, basal-like, and claudin-low breast cancers. These subtypes respond differently to therapy and are associated with different outcomes, with the shortest survival times seen in patients with basal-like and HER2-overexpressing subtypes [4,5,9].

Triple-negative breast cancer (TNBC) is an aggressive clinical phenotype characterized by the lack of expression (or minimal expression) of the estrogen receptor (ER) and progesterone receptor (PR) as well as the absence of the human epidermal growth factor receptor-2 (HER2). TNBCs comprise a heterogeneous subgroup of tumors, including but not limited to those classified by expression profiling as basal-like and claudin-low subtypes. TNBCs account for about 15% of all breast cancers [7,8,9,10]. Unlike patients suffering from ER/PR-positive or HER2-overexpressing cancers, treatment options for patients with TNBC are currently limited to systemic cytotoxic chemotherapy [11]. The overall survival rates of TNBC patients are lower than those of patients suffering from other phenotypes of breast cancer (in both early and advanced stages) [12,13]. These facts highlight the urgent need for effective medicines for the treatment of TNBCs.

Currently, compounds targeting the vascular endothelial growth factor (VEGF), poly (ADP-ribose) polymerase (PARP), HSP90, and aurora kinase are under investigation in clinical trials as therapeutics for metastatic TNBCs [14]. Herein, we report the computer-guided discovery of *N*-(naphtho[1,2-*b*]furan-5-yl)benzenesulfonamides as effective and selective inhibitors of TNBCs. These compounds serve as starting points for the development of effective drugs.

## 2. Results and Discussion

### 2.1. Three-Dimensional Similarity Search and Bioassays

**Figure 1:**
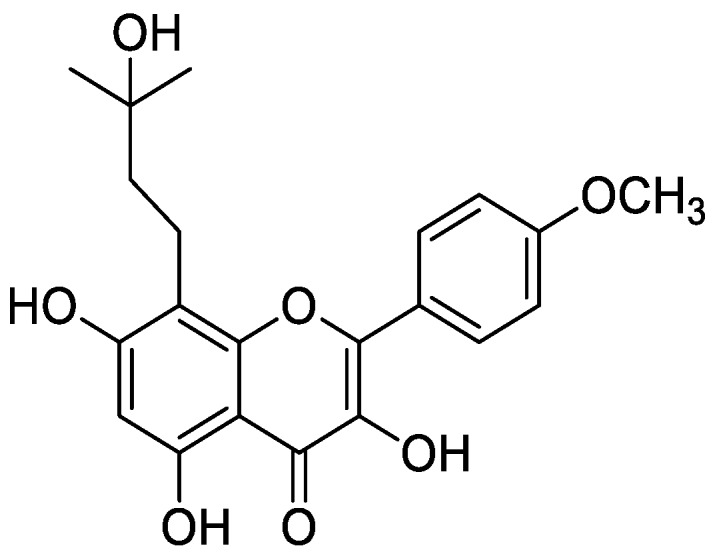
Chemical structure of **IC-163**.

**Figure 2:**
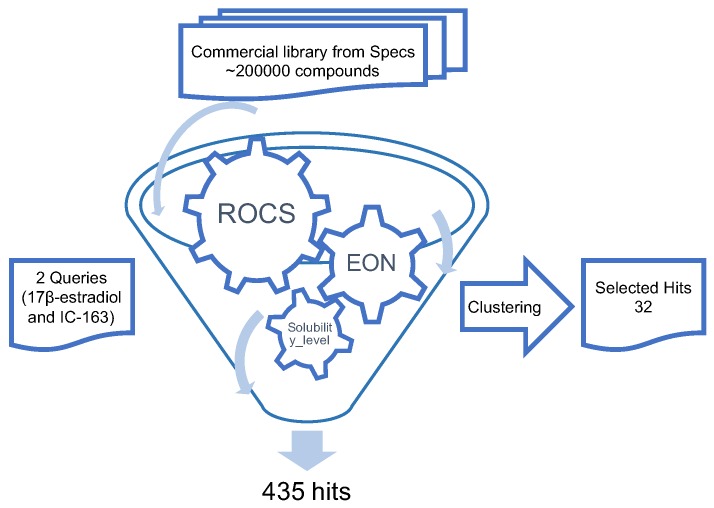
Scheme for three-dimensional virtual screening.

Estrogens are known to stimulate cell proliferation and increase the risk of the development of several different types of cancers, in particular breast and uterus cancers [15]. In order to identify novel inhibitors of breast cancers, we employed similarity-based computational approaches to search the SPECS compound library (http://www.specs.net/, accessed by May 2014) for candidate compounds. 17β-estradiol and **IC-163** (Figure 1), a potential agent for breast cancer identified by Beijing Shenogen Biomedical Co. [16], were chosen as query molecules for 3D similarity search (Figure 2).

In total, approximately 200 k compounds were screened with ROCS, an alignment-based virtual screening engine quantifying the similarity of pairs of molecules based on their molecular shapes and chemical features [17,18]. The most interesting compounds (selected by visual inspection) were re-ranked with EON (version 2.2.0, OpenEye Scientific Software Inc., Santa Fe, NM, USA) to evaluate compound similarity with regard to electrostatics. EON quantifies the similarity between pairs of molecules based on their electrostatic potential maps. Comparison with EON resulted in rank-ordered list of 435 candidate molecules, which was further reduced by clustering with ECFP_6 and FCFP_6 fingerprints. In total, 32 candidate compounds (Figure S1) were selected by visual inspection (taking into account calculated aqueous solubility) and purchased from SPECS for experimental evaluation. Twenty-five of the selected compounds originate from 17β-Estradiol as query and seven from **IC-163**.

**Figure 3:**
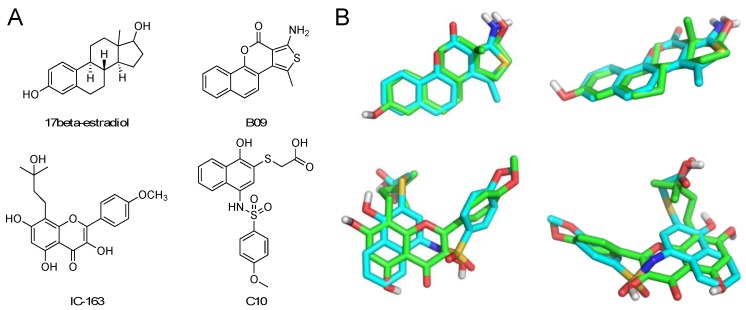
(**A**) Structures of 17β-estradiol, **B09**, **IC-163** and **C10**; (**B**) Structure alignment of **B09** with 17β-estradiol and of **C10** with **IC-163**.

The inhibition rates of these 32 compounds were measured on the TNBC cell lines MDA-MB-231 and SUM-159, as well as the non-TNBC breast cancer cell line MCF-7 (Figure S2). The most interesting compounds identified among those 32 candidates were **B09** and **C10**. Compound **B09**, identified by similarity search using 17β-estradiol as query, inhibited MCF-7 cells with IC_50_ = 1.45 μM and had almost no growth-inhibitory effect on MDA-MB-231 and SUM-159. 17β-Estradiol is an endogenous molecule directly interacting with the estrogen receptor. This may explain why **B09** only inhibited MCF-7 cells. **B09** is structurally related to 17β-estradiol not only with respect to its 3D shape but also its 2D structure (Figure 3). On the contrary, **C10** showed good inhibition of TNBC cell lines (IC_50_ = 2.32 μM for MDA-MB-231; IC_50_ = 3.45 μM for SUM-159) but low inhibition of MCF-7 cells (IC_50_ = 20 μM; Table 1**)**. Its chemical structure is similar to that of **IC-163** with respect to the 3D molecular shape and electrostatic properties (ShapeTanimoto coefficient = 0.817; EON_ShapeTanimoto coefficient = 0.784, where values of 1 denote compounds with identical properties) but not with respect to the 2D structure (Figure 3).

### 2.2. Hit Follow-up and Expansion

2D similarity search based on ECFP_6 and FCFP_6 was conducted to identify further purchasable analogs of **C10** for experimental evaluation. A total of 12 analogs of **C10** were purchased from SPECS and tested on MDA-MB-231 and SUM-159 cell lines. The measured inhibition rates are reported in Figure S3. All compounds were initially tested only with two TNBC cell lines for cell viability at 5 μg/mL. Following this test, the inhibitory activity of any compounds with inhibition rates above 30% at 5 μg/mL (eight compounds) were tested on four TNBC cell lines and one non-TNBC cell line MCF-7 (Table 2). All eight compounds inhibited MDA-MB-231 and MDA-MB-453 cells with IC_50_ values lower than 10 μM. Most compounds inhibited SUM-159 and BT-20 cells with IC_50_ values greater than 10 μM; seven of these compounds inhibited MCF-7 cells with IC_50_ values greater than 40 μM. Among them, Compounds **2-5** and **2-8** exhibited the strongest inhibitory effect on all tested TNBC cell lines and had no inhibitory effect on MCF-7.

**Figure 4:**
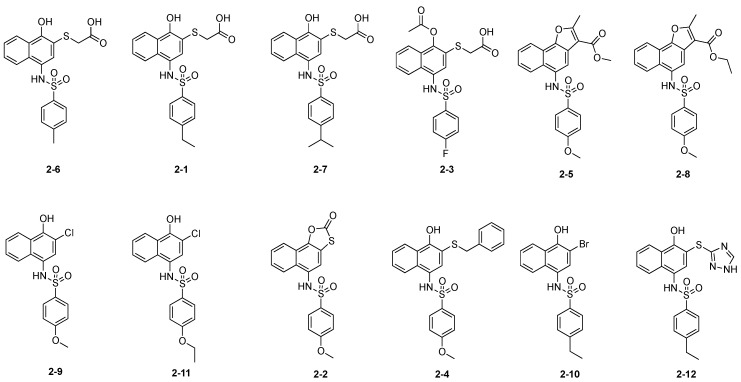
Structures of 12 compounds resulting from a similarity search based for **C10**.

All these compounds are based on a *N*-(naphthalen-1-yl)benzenesulfonamide scaffold (Figure 4), with different decorations in *para* position of the benzenesulfonamide and/or the substituent at the naphthalene ring. Compounds **C10**, **2-1**, **2-3**, **2-6**, and **2-7** are known inhibitors of myeloid cell leukemia 1 (Mcl-1) [19]. Compound **2-11** is an antimalarial heme detoxification protein (HDP) inhibitor [20], and **2-9** is an antitumor agent with inhibition of signal transducer and activator of transcription 3 (STAT3) [21,22]. We did not identify literature on the bioactivity of **2-5** and **2-8**. In terms of molecular structure, **2-5** and **2-8** clearly differ from other compounds of that series. They are decorated with a naphtho[1,2-*b*]furan in *para* position of the benzenesulfonamide. Compound **2-5** is a methyl carboxylate, while **2-8** is an ethyl carboxylate. Due to structural novelty and good bioactivity, **2-5** and **2-8** were selected for another iteration of hit expansion based on 2D similarity search.

A total of 40 analogs of **2-5** and **2-8** were purchased from SPECS and tested on MDA-MB-231, SUM-159, and MCF-7 cells (Figure S4). Twenty-four of these analogs did not exhibit activity (any compounds with an inhibition rate above 50% were considered to be active). One compound (**3-12**) inhibited all three of the cell lines, while **3-9**, **3-17**, **3-18**, **3-20**, and **3-37** only had an effect on two TNBC cell lines. Eleven compounds inhibited one TNBC cell line. Compounds with substitutions in *para* position of the benzenesulfonamide tended to be more active (Table 3 and Table 4). All tested compounds with R^3^ = methyl and R^1^ = ethoxy were active against TNBC, regardless of the length of R^2^. Compounds with a large substituent in R^2^ tended to be less active on SUM-159 cells. Compounds with R^1^ = ethyl were more active on MDA-MB-231 with R^2^ = methoxy than R^2^ = ethoxy. Compounds with a methoxy substituent in R^2^ were inactive on SUM-159. Replacement of the methyl moiety at R^3^ by a phenyl ring (e.g., **3-11** to **3-22**) or other groups (e.g., **2-8** to **3-28**) did not result in substantial changes in activity against the three cell lines, indicating that the substituent at the R^3^ position is likely less relevant for TNBC inhibition. Compounds with an isopropyl or chlorine substituent in R^1^ showed low activity on MDA-MB-231 and no activity on SUM-159 cells. When (1) R^3^ was methyl, (2) R^2^ was methoxy or ethoxy and (3) R^1^ was methyl, ethyl, methoxy, ethoxy, or fluorine, almost all compounds of this combination had different levels of inhibitory activities on both TNBC cell lines.

Compounds with more than one substituent or a fused ring system in the position of the phenyl ring of the benzenesulfonamide tended to have poor bioactivity (Table 4). The compounds available from SPECS and tested within the scope of this study only cover two or three methyl groups at different position of the benzene; other substituent groups like two or three methoxy groups were not measured. In addition, a compound including a tetracycline moiety (**3-21**) exhibited a low inhibitory activity on TNBC cells (Table S1).

**Scheme 1:**
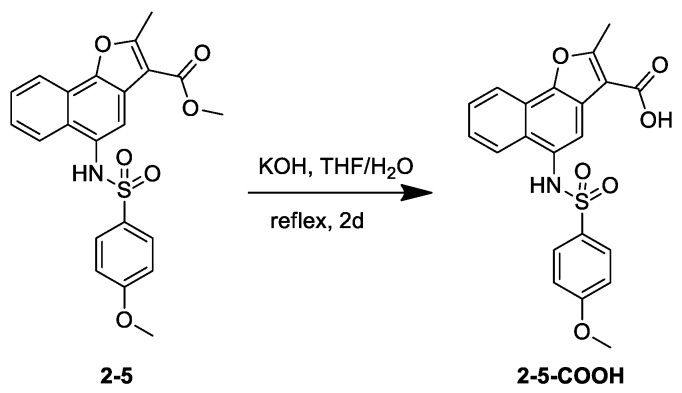
Hydrolysis route of Compound **2-5** to Compound **2-5-COOH**.

Ten compounds with good inhibition rates on both TNBC cell lines were selected for the measurement of IC_50_ values on MDA-MB-231 and SUM-159 cell lines. However, the aqueous solubility of these compounds is poor. Hydrolysis of the ester in the R^2^ position is expected to result in improved water solubility while maintaining biological activity. Therefore, we hydrolyzed **2-5** by refluxing it for two days under alkaline condition in the presence of an aqueous solution with potassium hydroxide and tetrahydrofuran (Scheme 1) to obtain **2-5-COOH**.

Most of the tested compounds inhibited both TNBC cell lines with similar strength (Table 5). Compound **2-5-COOH** had an approximately 10-fold reduced inhibitory activity compared to the ester compound (**2-5**). Its IC_50_ values were greater than 20 μM for both cell lines.

Seven of the most potent compounds were chosen for evaluation of their bioactivity in further cell lines. In addition to three TNBC cell lines (MDA-MB-231, MDA-MB-436, and SUM-159) and the breast cancer cell line MCF-7, we employed the normal mammary epithelial cell line MCF-10A, which was used for testing the compounds for their effects on normal breast cells (Table 6). The results show that most of the compounds exhibited a weak inhibition of MCF-10A cells. Compound **3-17**, the most potent TNBC inhibitor identified in this study, also had the strongest inhibition on MCF-10A (IC_50_ = 0.66 μM). Thus, **3-17** may be less promising for the treatment of TNBC.

**Figure 5:**
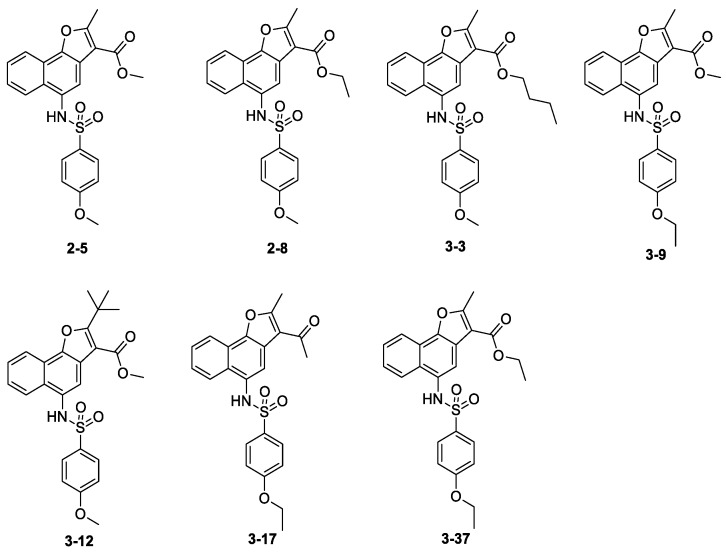
Structures of compounds in Table 6.

From a molecular structure point of view, the main differences of these compounds are the substituents of the phenyl ring of the benzenesulfonamide (**2-5**, **2-8**, **3-3**, and **3-12** are methoxy; **3-9**, **3-17**, and **3-37** are ethoxy), and/or naphtho[1,2-*b*]furan (Figure 5). Six compounds are esters, and Compound **3-17** is a ketone. Calculations suggest that **3-3** and **3-12** are less soluble in water, probably because of their longer carbon alkyl substituents (Table 6).

## 3. Materials and Methods

### 3.1. Overall Protocol

**Figure 6:**
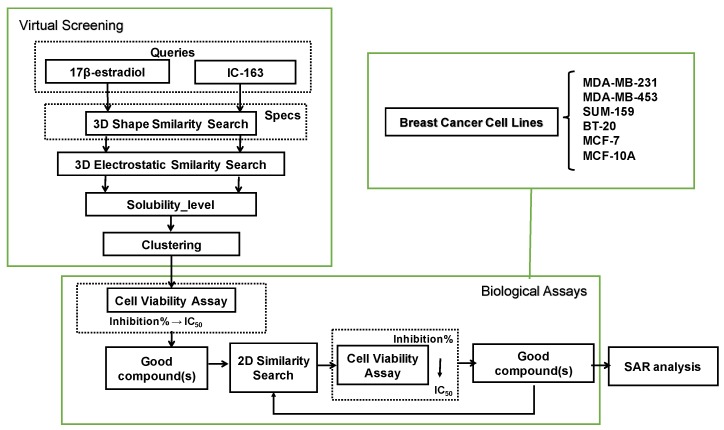
Workflow of virtual screening and bioassays.

3D screening methods taking into account the molecular shape and electrostatic maps of molecules were employed to identify compounds of interest in the SPECS compound library. Compounds were selected for purchase and experimental testing in cell-based assays taking into account their calculated aqueous solubility and structural diversity (assessed by a cluster analysis). An iterative 2D similarity search was conducted to follow up on active compounds and identify (further) derivatives for testing (Figure 6).

### 3.2. Three-Dimensional Similarity Search

The SPECS compound library (http://www.specs.net/, accessed May 2014) was prepared with a workflow developed with Pipeline Pilot v7.5 (PP 7.5, Accelrys Software, Inc., San Diego, CA, USA.), in which minor salt components were removed and the chemical structures standardized. The prepared database was filtered with “Blockbuster” filter of FILTER (version 2.2.1, OpenEye Scientific Software, Inc., Santa Fe, NM, USA) to remove molecules with undesired physicochemical properties with respect to molecular weight, number of heavy atoms, and aqueous solubility. Next, the databases were processed with OMEGA [23] (version 2.4.5, OpenEye Scientific Software, Inc., Santa Fe, NM, USA) to generate up to 500 conformations for each molecule.

ROCS [17,24] (version 3.2.0, OpenEye Scientific Software Inc., Santa Fe, NM, USA) was employed for 3D shape comparison. The lowest energy conformers of 17β-estradiol and **IC-163** generated with OMEGA served as input for screening with ROCS. EON was employed to re-rank the top-ranked molecules obtained with ROCS based on the similarity of electrostatic properties.

### 3.3. Water Solubility Prediction and Structure Cluster Analysis

Water solubility at 25 °C was calculated with the ADMET solubility prediction module of Discovery Studio 2.5 (Accelrys Software, Inc., San Diego, CA, USA). We removed molecules with ADMET solubility level in 0 (extremely low) and 1 (very low) to ensure that the chosen compounds have acceptable solubility. The remaining compounds were clustered based on ECFP_6 and FCFP_6 fingerprints to assist the selection of compounds for experimental testing.

### 3.4. Two-Dimensional Similarity Search

The most promising compounds, **C10**, **2-5**, and **2-8**, served as templates for 2D similarity search based on ECFP_6 or FCFP_6 fingerprints.

### 3.5. Cell Viability Assays

Four human TNBC cell lines (MDA-MB-231, MDA-MB-453, SUM-159, and BT-20), a non-TNBC breast cancer cell line (MCF-7) and a normal mammary epithelial cell line (MCF-10A) were cultured with DMEM (phenol free) supplemented with 2.5% CS-FBS and 1% l-Glu. 1.2 × 10^3^ of cells were seeded into 384-well microplates and maintained for 24 h in an incubator at 37 °C in a 5% CO_2_, saturated humidified atmosphere. Different compounds were added into cells with 9 concentrations from 0.156 to 40 μM for 72 h. Tamoxifen was the positive control. Then CCK-8 solution was added into cells and incubated for another 4 h. Absorbance was measured with a Microplate Reader at 450/600 nm. The IC_50_ values of the compounds on TNBC cell lines were derived using GraphPad Prism 5.0 (GraphPad Software, Inc., La Jolla, CA, USA).

## 4. Conclusions

3D and 2D similarity searches led to the identification of *N*-(naphtho[1,2-*b*]furan-5-yl)benzenesulfonamides as novel inhibitors of TNBCs. The most potent compounds (**2-5** and **2-8**) obtained IC_50_ values of 2–3 μM on different TNBC cell lines and showed no inhibitory activity on normal (i.e. MCF-7 and MCF-10A) cells in vitro, indicating their selectivity against TNBC cells. These compounds and derivatives thereof could serve as starting points for further research and development of selective TNBC inhibitors.

## Figures and Tables

**Table 1:** Activities of **B09** and **C10** measured on different cell lines.

Compound	IC_50_ (μM)		
	MDA-MB-231	SUM-159	MCF-7
B09	~20	>40	1.45
C10	2.32	3.45	20

**Table 2:** Activities of eight analogs of **C10** measured on different cell lines.

Compound	IC_50_ (μM)				
	MDA-MB-231	MDA-MB-453	SUM-159	BT-20	MCF-7
**Tamoxifen**	2.03	3.64	13.48	8.54	9.08
**IC-163**	>40	>40	>40	>40	>40
**C10**	5.34	2.30	11.13	10.63	9.38
**2-1**	4.36	3.19	30.76	13.34	13.97
**2-3**	7.99	8.59	>40	>40	>40
**2-5**	3.12	2.95	2.91	>40	>40
**2-6**	4.97	3.36	19.21	25.02	>40
**2-7**	3.95	3.65	>40	>40	>40
**2-8**	2.96	3.09	2.50	10.10	>40
**2-9**	6.22	4.61	18.06	>40	>40
**2-11**	6.92	5.72	>40	>40	>40

**Table 3:**
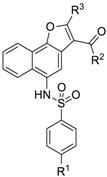
Inhibition rates of analogs of **2-5** and **2-8** identified by 2D similarity search (Part 1).

Compound	R^1^	R^2^	R^3^	Inhibition%		
				MDA-MB-231	SUM-159	MCF-7
**3-17**	-OCH_2_CH_3_	-CH_3_	-CH_3_	68.567	60.956	40.549
**3-8**	-CH_2_CH_3_	-OH	-CH_3_	−1.131	0.221	−6.706
**3-32**	-CH(CH_3_)_2_	-OH	-CH_3_	24.718	−0.964	−7.568
**3-1**	-Cl	-OH	-CH_3_	31.450	−0.754	−12.178
**3-9**	-OCH_2_CH_3_	-OCH_3_	-CH_3_	63.008	60.778	24.251
**3-37**	-OCH_2_CH_3_	-OCH_2_CH_3_	-CH_3_	59.687	66.789	42.503
**3-23**	-OCH_2_CH_3_	-OCH_2_CH_2_OCH_3_	-CH_3_	62.014	49.127	14.707
**3-20**	-CH_2_CH_3_	-OCH_3_	-CH_3_	53.486	65.439	31.221
**3-11**	-CH_2_CH_3_	-OCH_2_CH_3_	-CH_3_	41.115	0.175	13.005
**3-22**	-CH_2_CH_3_	-OCH_2_CH_3_	-Ph	53.616	−0.556	2.238
**3-4**	-CH(CH_3_)_2_	-OCH_3_	-CH_3_	37.770	−0.319	14.222
**3-31**	-CH(CH_3_)_2_	-OCH_2_CH_3_	-CH_3_	32.074	−1.330	2.952
**3-30**	-H	-OCH_3_	-CH_3_	50.872	0.862	29.311
**3-14**	-CH_3_	-OCH_3_	-CH_3_	62.843	48.214	10.194
**2-5**	-OCH_3_	-OCH_3_	-CH_3_	31.005	56.793	-
**3-10**	-F	-OCH_3_	-CH_3_	64.477	35.466	34.480
**3-33**	-Cl	-OCH_3_	-CH_3_	32.822	−0.507	2.819
**3-29**	-Br	-OCH_3_	-CH_3_	45.039	1.3243	14.480
**3-34**	-COOH	-OCH_3_	-CH_3_	10.991	−0.042	2.848
**3-26**	-H	-OCH_2_Ph	-CH_3_	51.036	1.581	25.556
**3-36**	-CH_3_	-OCH_2_CH_3_	-CH_3_	16.752	−0.123	−4.385
**2-8**	-OCH_3_	-OCH_2_CH_3_	-CH_3_	62.936	61.771	-
**3-3**	-OCH_3_	-OCH_2_CH_2_CH_2_CH_3_	-CH_3_	51.145	42.064	29.745
**3-25**	-OCH_3_	-OCH_2_CH_2_CH_2_CH_2_CH_3_	-CH_3_	39.505	61.902	48.321
**3-28**	-OCH_3_	-OCH_2_CH_3_	-CH_2_CH_2_CH_3_	61.911	46.000	33.448
**3-12**	-OCH_3_	-OCH_3_	-C(CH_3_)_3_	71.378	90.386	76.319
**3-39**	-F	-OCH(CH_3_)_2_	-CH_3_	42.330	−0.193	4.948
**3-13**	-COOH	-OCH_2_CH_3_	-CH_3_	19.635	−0.635	5.137
**3-40**	-COOH	-OCH_2_CH_3_	-CH_2_CH_2_CH_3_	12.487	0.000	2.525

**Table 4:**
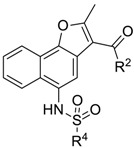
Inhibition rates of analogs of **2-5** and **2-8** identified by 2D similarity search (Part 2).

Compound	R^4^	R^2^	Inhibition%		
			MDA-MB-231	SUM-159	MCF-7
**3-27**	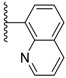	-OCH_3_	48.153	10.205	35.546
**3-7**	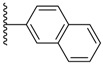	-OCH_3_	0.431	0.085	−4.733
**3-6**	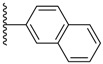	-OCH_2_CH_3_	43.365	0.292	7.724
**3-5**	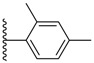	-OCH_3_	41.065	0.757	21.98
**3-15**	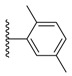	-OCH_3_	45.584	0.037	18.546
**3-38**	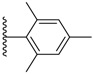	-OCH_3_	50.558	1.346	14.827
**3-24**	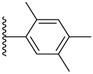	-OCH_3_	46.563	0.233	34.910
**3-16**	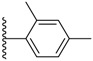	-OCH_2_CH_3_	48.171	0.732	43.439
**3-35**	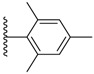	-OCH_2_CH_3_	33.853	0.352	24.499
**3-2**	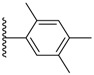	-OCH_2_CH_3_	24.255	−0.884	17.700
**3-19**	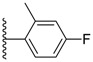	-OH	19.306	0.380	16.340
**3-18**	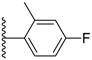	-OCH_3_	59.421	51.425	33.244

**Table 5:** IC_50_ for compounds measured on two TNBC cell lines.

Compound	IC_50_ (μM)	
	MDA-MB-231	SUM-159
2-5	3.12	2.91
2-8	2.96	2.50
3-3	3.25	8.66
3-9	2.68	2.66
3-12	11.91	11.53
3-17	1.77	1.94
3-18	5.20	24.15
3-20	10.04	16.97
3-28	>40	14.41
3-37	5.49	4.09
2-5-COOH	~24	24.25

**Table 6:** IC_50_ of compounds measured on different cell lines and calculated molecular properties.

Compound	IC_50_ (μM)					Calculated Molecular Properties		
	MDA-MB-231	MDA-MB-436	SUM-159	MCF-7	MCF-10A	Molecular_Weight	AlogP	ADMET_Solubility_level
**Tamoxifen**	2.03	10.02	13.48	9.08	>40	371.5	6.319	1
**2-5**	3.12	1.99	2.91	>40	>40	425.5	3.514	2
**2-8**	2.96	2.80	2.50	>40	>40	439.5	3.863	2
**3-3**	3.25	3.36	8.66	ND ^a^	>40	467.5	4.842	1
**3-9**	2.68	2.08	2.66	ND	>40	439.5	3.863	2
**3-12**	11.91	7.13	11.53	ND	>40	467.5	5.063	1
**3-17**	1.77	~0.01	1.94	ND	0.66	423.5	3.747	2
**3-37**	5.49	1.41	4.09	ND	> 40	453.5	4.211	2

ND ^a^: not determined.

## Supplementary Materials

The following are available online. Figure S1: Structures of 32 compounds from 3D similarity search; Figure S2: Inhibition rates of 32 compounds at 10 μM from 3D similarity search; Figure S3: Inhibition rates of 12 compounds at 5 μg/mL from C10 2D similarity search; Figure S4: Inhibition rates of 40 compounds at 5 μg/mL from Compounds **2-5** and **2-8** 2D similarity search; Table S1: Inhibition rates of analogs of **2-5** and **2-8** identified by 2D similarity search (Part 3).

## Abbreviations

TNBC: triple-negative breast cancer
ER: estrogen receptor
PR: progesterone receptor
HER2: human epidermal growth factor receptor 2
VEGF: the vascular epidermal growth factor
3D: three-dimensional
ROCS: Rapid Overlay of Chemical Structures
2D: two-dimensional
ECFP_6: extended connectivity fingerprints of maximum diameter 6
FCFP_6: function class fingerprints of maximum diameter 6

## References

[1] Siegel R.L., Miller K.D., Jemal A. (2016). Cancer statistics, 2016. CA Cancer J. Clin..

[2] Fan L., Strasser-Weippl K., Li J.J., St Louis J., Finkelstein D.M., Yu K.D., Chen W.Q., Shao Z.M., Goss P.E. (2014). Breast cancer in China. Lancet Oncol..

[3] Perou C.M., Sørlie T., Eisen M.B., van de Rijn M., Jeffrey S.S., Rees C.A., Pollack J.R., Ross D.T., Johnsen H., Akslen L.A. (2000). Molecular portraits of human breast tumours. Nature.

[4] Sørlie T., Perou C.M., Tibshirani R., Aas T., Geisler S., Johnsen H., Hastie T., Eisen M.B., van de Rijn M., Jeffrey S.S. (2001). Gene expression patterns of breast carcinomas distinguish tumor subclasses with clinical implications. Proc. Natl. Acad. Sci. USA.

[5] Sørlie T., Tibshirani R., Parker J., Hastie T., Marron J., Nobel A., Deng S., Johnsen H., Pesich R., Geisler S. (2003). Repeated observation of breast tumor subtypes in independent gene expression data sets. Proc. Natl. Acad. Sci. USA.

[6] Uscanga-Perales G.I., Santuario-Facio S.K., Ortiz-López R. (2016). Triple negative breast cancer: Deciphering the biology and heterogeneity. Med. Univ..

[7] Hennessy B.T., Gonzalez-Angulo A.-M., Stemke-Hale K., Gilcrease M.Z., Krishnamurthy S., Lee J.-S., Fridlyand J., Sahin A., Agarwal R., Joy C. (2009). Characterization of a naturally occurring breast cancer subset enriched in epithelial-to-mesenchymal transition and stem cell characteristics. Cancer Res..

[8] Herschkowitz J.I., Simin K., Weigman V.J., Mikaelian I., Usary J., Hu Z., Rasmussen K.E., Jones L.P., Assefnia S., Chandrasekharan S. (2007). Identification of conserved gene expression features between murine mammary carcinoma models and human breast tumors. Genome Biol..

[9] Onitilo A.A., Engel J.M., Greenlee R.T., Mukesh B.N. (2009). Breast cancer subtypes based on ER/PR and Her2 expression: Comparison of clinicopathologic features and survival. Clin. Med. Res..

[10] Prat A., Adamo B., Cheang M.C., Anders C.K., Carey L.A., Perou C.M. (2013). Molecular characterization of basal-like and non-basal-like triple-negative breast cancer. Oncologist.

[11] O’Reilly E.A., Gubbins L., Sharma S., Tully R., Guang M.H.Z., Weiner-Gorzel K., McCaffrey J., Harrison M., Furlong F., Kell M. (2015). The fate of chemoresistance in triple negative breast cancer (TNBC). BBA Clin..

[12] Dent R., Trudeau M., Pritchard K.I., Hanna W.M., Kahn H.K., Sawka C.A., Lickley L.A., Rawlinson E., Sun P., Narod S.A. (2007). Triple-negative breast cancer: Clinical features and patterns of recurrence. Clin. Cancer Res..

[13] Haffty B.G., Yang Q., Reiss M., Kearney T., Higgins S.A., Weidhaas J., Harris L., Hait W., Toppmeyer D. (2006). Locoregional relapse and distant metastasis in conservatively managed triple negative early-stage breast cancer. J. Clin. Oncol..

[14] Mancini P., Angeloni A., Risi E., Orsi E., Mezi S. (2014). Standard of care and promising new agents for triple negative metastatic breast cancer. Cancers.

[15] Suba Z. (2014). Triple-negative breast cancer risk in women is defined by the defect of estrogen signaling: Preventive and therapeutic implications. OncoTargets Ther..

[16] Li J., Meng K. (2008). Compounds and Methods for Treating Estrogen Receptor-Related Diseases. U.S. Patent.

[17] Hawkins P.C., Skillman A.G., Nicholls A. (2007). Comparison of shape-matching and docking as virtual screening tools. J. Med. Chem..

[18] Rush T.S., Grant J.A., Mosyak L., Nicholls A. (2005). A shape-based 3-D scaffold hopping method and its application to a bacterial protein-protein interaction. J. Med. Chem..

[19] Abulwerdi F.A., Liao C., Mady A.S., Gavin J., Shen C., Cierpicki T., Stuckey J.A., Showalter H.D., Nikolovska-Coleska Z. (2014). 3-substituted-*N*-(4-hydroxynaphthalen-1-yl)arylsulfonamides as a novel class of selective Mcl-1 inhibitors: Structure-based design, synthesis, SAR, and biological evaluation. J. Med. Chem..

[20] Rathore D., Jani D., Nagarkatti R. (2007). HDP (Heme Detoxification Protein) Involved in Hemozoin Formation in Plasmodium and Theileria as an Anti-protozoal Target, and High-throughput Screening for Antimalarial HDP Inhibitors. U.S. Patent.

[21] Tweardy D.J., Huang X., Kasembeli M.M. (2009). Stat3 Inhibitors. W.O. Patent.

[22] Tweardy D.J., Kasembeli M.M., Xu M.X., Eckols T.K. (2015). Methods and Compositions for Treatment of Muscle Wasting, Muscle Weakness, and/or Cachexia Using Inhibitors of Stat3. W.O. Patent.

[23] Hawkins P.C.D., Skillman A.G., Warren G.L., Ellingson B.A., Stahl M.T. (2010). Conformer generation with OMEGA: Algorithm and validation using high quality structures from the protein databank and cambridge structural database. J. Chem. Inf. Model..

[24] Grant J.A., Gallardo M., Pickup B.T. (1996). A fast method of molecular shape comparison: A simple application of a gaussian description of molecular shape. J. Comput. Chem..

