# Supplementary material for: Discovery of N-(Naphtho[1,2-b]Furan-5-Yl) Benzenesulfonamides as Novel Selective Inhibitors of Triple-Negative Breast Cancer (TNBC)
Source: Molecules. 2018 Mar 16;23(3):678. doi: 10.3390/molecules23030678 (PMC6017705; doi:10.3390/molecules23030678)
Supplement: Supplementary file 1 [file molecules-23-00678-s001.pdf]

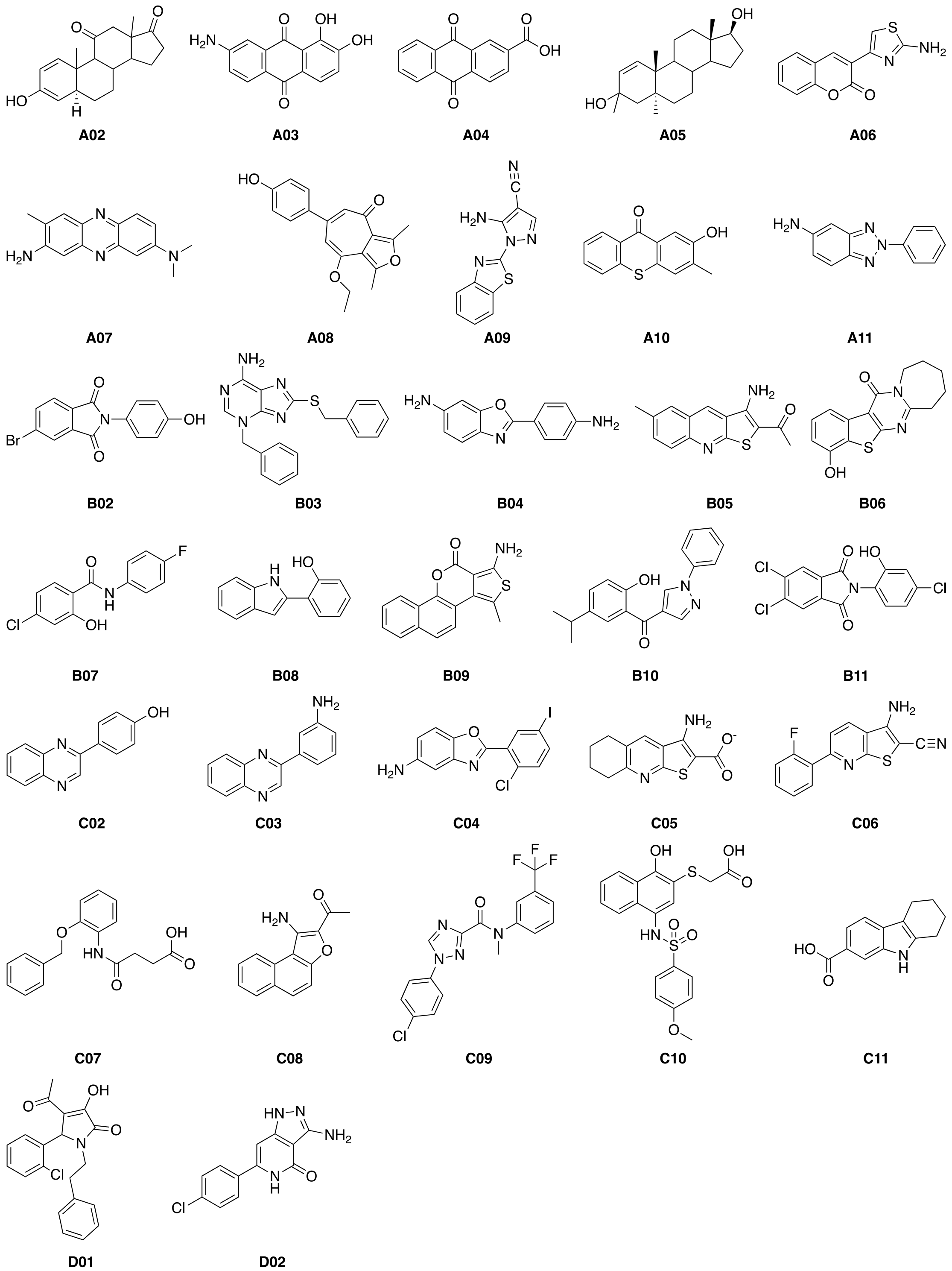

Figure S1. Structures of 32 compounds from 3D similarity search.

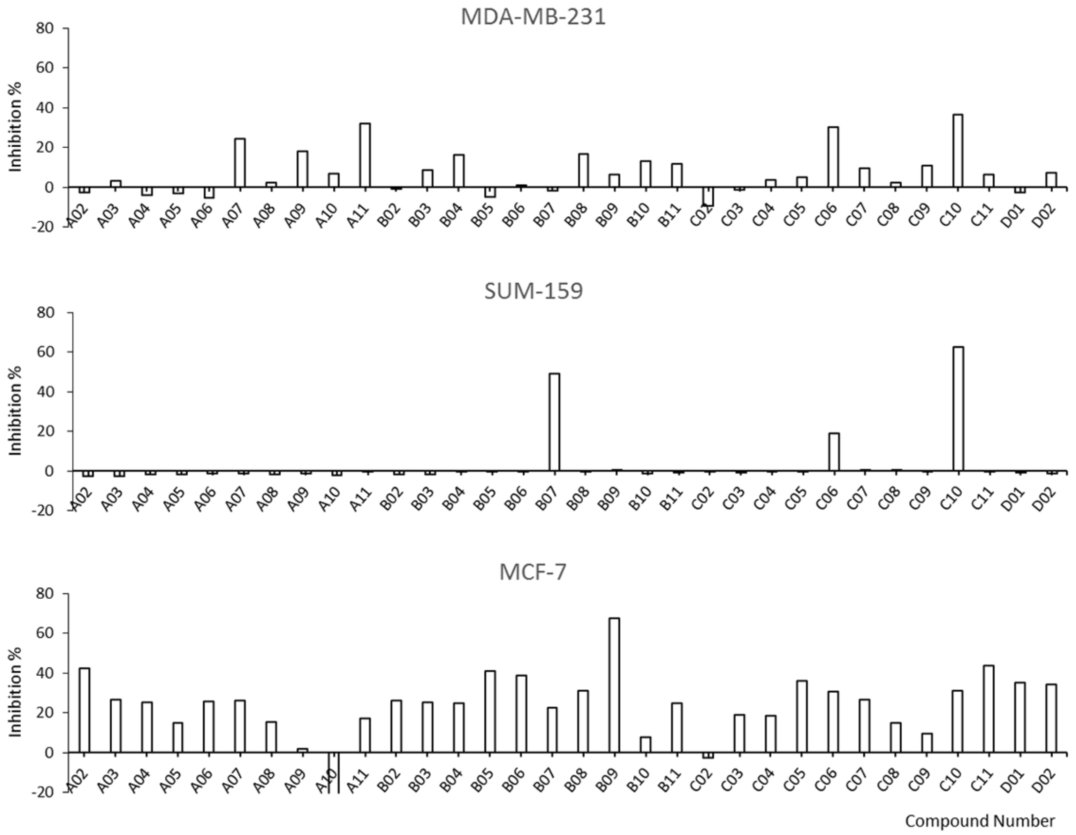

Figure S2. Inhibition rates of 32 compounds at 10 μM from 3D similarity search.

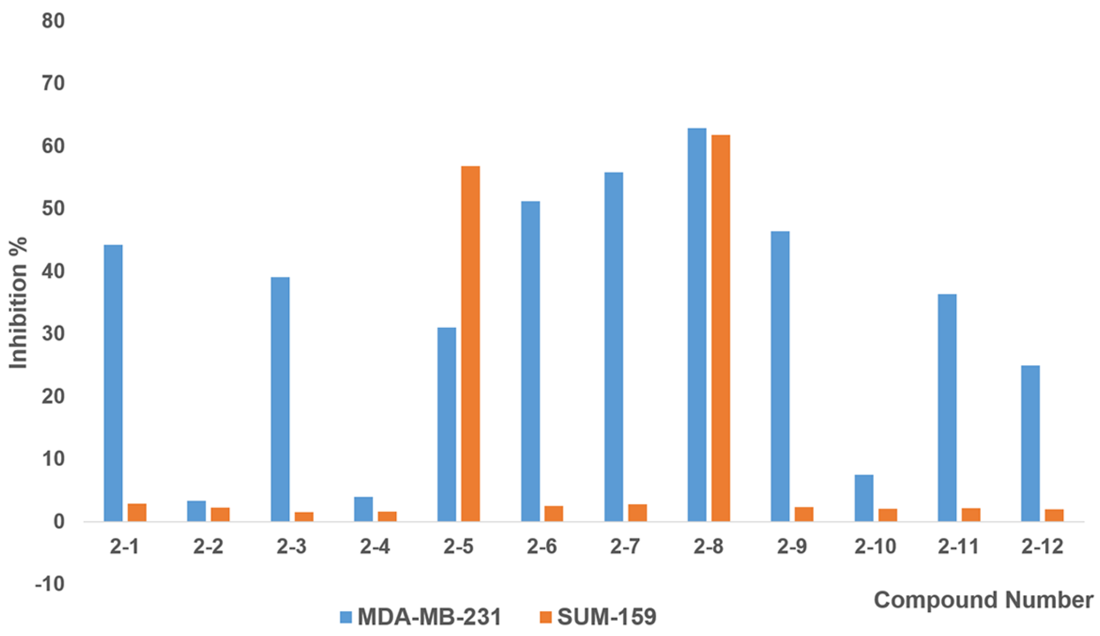

Figure S3. Inhibition rates of 12 compounds at 5 μg/ml from **C10** 2D similarity search.

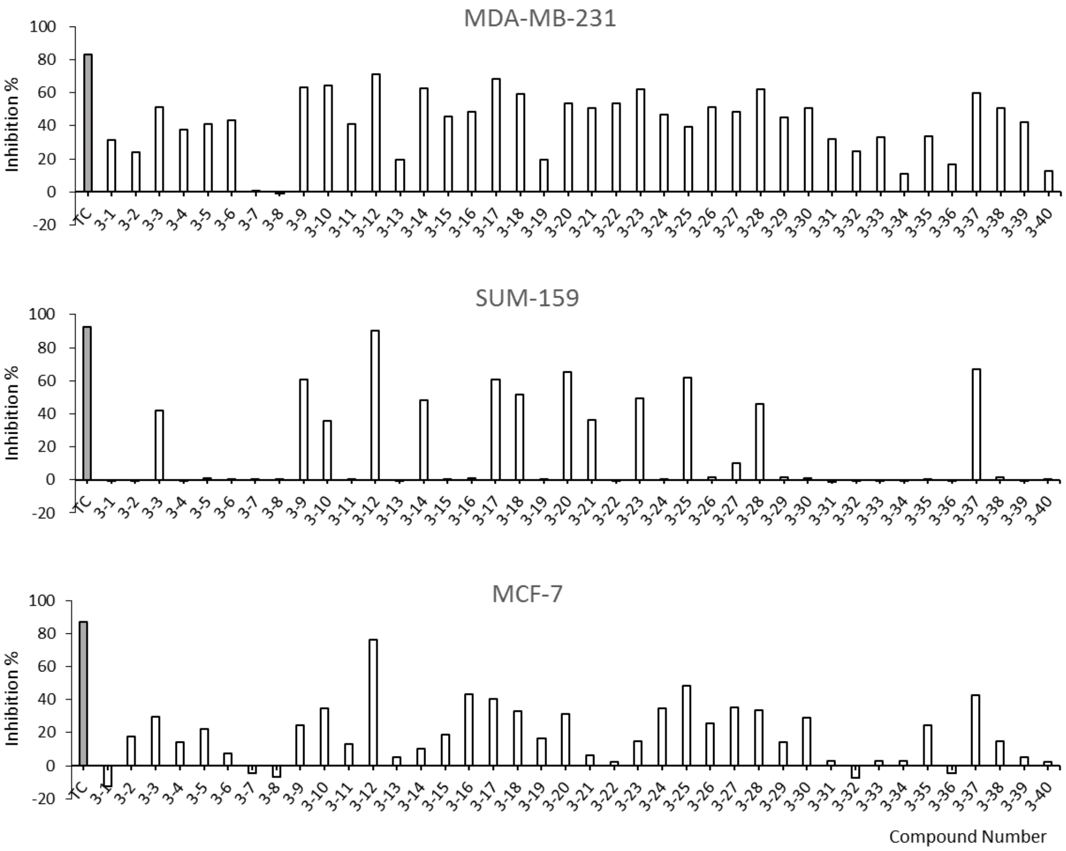

Figure S4. Inhibition rate of 40 compounds at 5 μg/ml from **2-5** and **2-8** 2-D similarity search.

Table S1. Inhibition rates of analogs of **2-5** and **2-8** 2D similarity search (Part3).

**Compound**	**Structure**	**Inhibition%**		
		MDA-MB-231	SUM-159	MCF-7
**3-21**	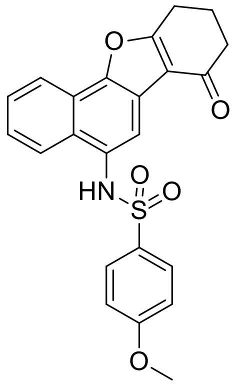	50.511	36.385	6.218
